# Mapping Disease at an Approximated Individual Level Using Aggregate Data: A Case Study of Mapping New Hampshire Birth Defects

**DOI:** 10.3390/ijerph10094161

**Published:** 2013-09-06

**Authors:** Xun Shi, Stephanie Miller, Kevin Mwenda, Akikazu Onda, Judy Rees, Tracy Onega, Jiang Gui, Margaret Karagas, Eugene Demidenko, John Moeschler

**Affiliations:** 1 Department of Geography, Dartmouth College, 6017 Fairchild, Hanover, NH 03755, USA; E-Mail: akikazu.onda.12@dartmouth.edu; 2 The Children’s Environmental Health and Disease Prevention Center, The Geisel School of Medicine at Dartmouth, Lebanon, NH 03766, USA; E-Mails: stephanie.d.miller@hitchcock.org (S.M.); judith.r.rees@dartmouth.edu (J.R.); tracy.l.onega@dartmouth.edu (T.O.); jiang.gui@dartmouth.edu (J.G.); margaret.r.karagas@dartmouth.edu (M.K.); eugene.demidenko@dartmouth.edu (E.D.); john.moeschler@dartmouth.edu (J.M.); 3 Department of Geography, University of California at Santa Barbara, Santa Barbara, CA 93106, USA; E-Mail: kmwenda@geog.ucsb.edu

**Keywords:** birth defects, aggregate data, disaggregation, Monte Carlo, disease mapping, New Hampshire

## Abstract

*Background:* Limited by data availability, most disease maps in the literature are for relatively large and subjectively-defined areal units, which are subject to problems associated with polygon maps. High resolution maps based on objective spatial units are needed to more precisely detect associations between disease and environmental factors. *Method:* We propose to use a *Restricted and Controlled Monte Carlo* (RCMC) process to disaggregate polygon-level location data to achieve mapping aggregate data at an approximated individual level. RCMC assigns a random point location to a polygon-level location, in which the randomization is *restricted* by the polygon and *controlled* by the *background* (e.g., population at risk). RCMC allows analytical processes designed for individual data to be applied, and generates high-resolution raster maps. *Results:* We applied RCMC to the town-level birth defect data for New Hampshire and generated raster maps at the resolution of 100 m. Besides the map of significance of birth defect risk represented by *p-*value, the output also includes a map of spatial uncertainty and a map of hot spots. *Conclusions:* RCMC is an effective method to disaggregate aggregate data. An RCMC-based disease mapping maximizes the use of available spatial information, and explicitly estimates the spatial uncertainty resulting from aggregation.

## 1. Introduction

Geospatial analysis can help form hypotheses and research designs for epidemiological and public health studies [1,2]. The first stage of this analysis is mapping the disease occurrence. Prior analyses have revealed considerable non-random spatial variation in occurrences of birth defects, raising suspicions of its associations with certain environmental factors [3,4,5,6,7,8,9,10,11,12,13,14,15]. However, most, if not all, of these previous studies used aggregate data to generate polygon maps for areal units (e.g., zip (postal) code, town, and census tract). This, in fact, reflects a general situation in disease mapping: Most disease maps in literature and in public health practice are based on aggregate data and are for fairly large areal units with irregular sizes and shapes, typically due to limited data accessibility related primarily to privacy issues. These maps thus are subject to some classic pitfalls that have been studied in spatial analysis, including:

(1) Modifiable areal unit problem (MAUP): The analysis result may be considerably affected by how the areal units are defined (e.g., [16,17]);
(2) Small number problem: The value for an area unit may be too small to be considered statistically stable (e.g., [1,2]);
(3) Unrealistic geographical assumptions: The mapping either assumes that the subjects are evenly distributed across the areal unit or all are concentrated on one point, e.g., the centroid of the polygon [18]; and
(4) Unidentifiable spatial uncertainty: While it is a known fact that data aggregation causes spatial uncertainty due to location imprecision (use a polygon to represent a point) and inaccuracy (use the polygon centroid to represent all locations in the polygon), with a conventional polygon map, there is no effective way to estimate, represent, and present this uncertainty [18,19].

Using a dataset of AIDS in Michigan, Jacquez and Waller [20] found that the result of a spatial cluster analysis based on centroids differs substantially from that based on individual locations, and the higher the aggregation level, the lower the statistical power of the analysis. An important implication of this kind of study is to use individual data whenever possible. Some recent studies on the impact of aggregation, including [21,22], generally confirm that aggregation may reduce statistical power and lead to inaccuracy in disease mapping. Research also shows that disaggregation based on auxiliary population information may improve the quality of disease mapping [21].

In this study, we mapped birth defects in New Hampshire using a *Restricted and Controlled Monte Carlo* (RCMC) method [18,19]. This method takes advantage of modern computing technology to disaggregate polygon-level data to approximated point level (pixel) so as to map disease at a much finer scale than what is allowed by directly using the original data for large areal units. In addition, the RCMC process converts location data from being based on irregularly-defined areal units to being based on regularly-defined units that have the same size and shape (pixels), mitigating the problems associated with irregularly-defined areal units. Compared with the published studies that adopted the RCMC method, in this study we further developed this method in two aspects:

(1) In the previous studies, this method was employed to deal with the situation that the location data is a mixture of point-level and polygon-level data (typically P.O. Box numbers), in which the primary function of RCMC is to disaggregate the polygon-level portion to make them compatible with the point-level data, so that the following disease mapping process (e.g., kernel density estimation or KDE) can be applied. In this study, we further pushed this method to the situation that the data are entirely aggregate in the first place, which is more common in disease mapping practice. By doing this, conceptually we took this method as a general approach to incorporating auxiliary information (e.g., detailed background population data) for the purpose of improving and evaluating spatial certainty of the data used for disease mapping, as well as mitigating the problems associated with mapping based on irregularly defined large areal units.
(2) In previous studies, the RCMC method was applied to a disease (lung cancer) that has a broad cohort (*i.e.*, not limited to a specific category in population), and therefore the background data (*expected count*) were able to be directly derived from the data of general population and represented as raster. Technically, in those studies the RCMC for disease cases and the following KDE were directly run over the raster backgrounds. Differently, the diseases we addressed in this study, birth defects, have a very specific cohort (infants) rather than general population. The location data of cohort are also aggregate and need to be disaggregated through RCMC. The disaggregation of disease cases and the following KDE need to be based on the disaggregated cohort locations, instead of directly on the general population (or its derivatives). In other words, instead of the case-background two-level structure in the previous studies, in this study we were dealing with a case-cohort-population three-level hierarchy. The extra layer of cohort brings about a great complexity to the implementation of the RCMC method.

## 2. Data

### 2.1. Birth Defect Data and All-Birth Data

Birth defect data were obtained from the New Hampshire Birth Conditions Program (NHBCP) for the period 2003–2009 (N = 2,289). Each record contained: (1) description of the defect; (2) information about the infant, including birth date, gestational age, sex, birth weight, plurality, and birth order; and (3) information about the mother, including age at delivery, race/ethnicity, and residential location as town and zip code. The NHBCP registry is a population-based surveillance system in which data are collected by the NHBCP through medical record reviews conducted at all NH hospitals and a sub-set of specialty care providers. The use of this dataset in this study was approved by the authoritative institutional review board (IRB). 

Prior to analysis, we processed the raw data in several ways. First, the raw data were based on birth defect occurrence, not by infants, and thus an infant with multiple defects has multiple records. Since our intention was to map the intensity of birth defects based on infants, we organized the data so that each infant would be represented only once. Second, we excluded records with unknown maternal age (~10% of records) because maternal age is an important potential covariate. Third, we excluded infants born to mother’s age > 49 years, due to the small numbers of both births defects and total births in this stratum. Fourth, we excluded multiple births (*i.e*., twins and triplets, accounting for about 9% of all the records). 

As our ultimate goal is to determine as yet unidentified environmental factors that may be associated with birth defects, we excluded fetal alcohol syndrome and chromosomal defects (*i.e*., Down syndrome, Trisomy 13, and Trisomy 18, accounting for about 8% of all the records), because these conditions result from known causes. The final database used for the analysis contains records of 1,395 infants.

To provide the “background” births, we obtained New Hampshire birth certificates for the same period (N = 101,435) from the New Hampshire Department of Human and Health Services (NH DHHS). This dataset included infant birth date, gestational age, birth weight, sex, mother’s age at delivery, and mother’s residential town and zip code at delivery. These records were filtered to exclude: (1) non-NH addresses, (2) multiple births, (3) mothers whose ages were either missing or outside the range of 15-49 years. The remaining births (N = 98,982) were used in the following analysis. This dataset is referred to as *all births* herein. The use of this dataset in this study was approved by the authoritative IRB.

Previous studies identify mother’s age and race/ethnicity as factors related to the risk of birth defects [23]. However, in our analysis we did not consider race/ethnicity, because on the one hand the birth certificates do not contain race/ethnicity information, and on the other hand, according to the Census 2010 data, non-white females aged 15–49 years only account for about 7% of the population in this age range in NH. Based on the age breaks of the Census data, we calculated the ratio of infants with defects to the total births for each age group. The results are listed in Table 1. 

**Table 1:** Birth defect ratio by mother’s age category, New Hampshire, 2003–2009.

Age	BD Infants	All Births	Ratio
**15–19**	83	5,909	0.0140
**20–24**	322	18,823	0.0171
**25–29**	391	26,369	0.0148
**30–34**	361	27,180	0.0133
**35–39**	180	13,625	0.0132
**40–44**	55	2,840	0.0194
**45–49**	*	<200	0.0208
**Two-Category Division:**			
**15–39**	1,337	91,906	0.0145
**40–49**	<100	<3,000	0.0194

* Data suppressed due to small value.

It appears that generally the ratios have a two-step pattern with a jump occurring at a maternal age of 40 years. Therefore, we adopted a two-category stratification: 15–39 and 40–49 years. We aggregated the more detailed age categories into two as a balance between age-specific precision and statistical and spatial certainties. While the mother’s place of residence was given by both town name and zip code, we chose to use town. Town and zip code are at similar spatial scales in NH, but the polygons of towns are more regular in terms of size and shape.

### 2.2. Spatially Detailed Population Data

For disaggregation, we created GIS data layers that detail the spatial distribution of women aged 15–49 years in NH. They are integrations of the LandScan^™^ data from Oak Ridge National Laboratory (ORNL) and the Census data to capitalize on complementary aspects of each of the two datasets. A major problem with the Census data is the lack of spatial details within units having large spatial extent and small population size, which is typical in rural areas. For example, the largest census block in NH is 331 km^2^ in size and has less than 300 people. An analysis solely using the Census data would have to assume that these people are either evenly distributed across the 331 km^2^ area or concentrated on a spot (e.g., centroid of the polygon), neither being realistic. The LandScan^™^ process mitigates this problem by allocating the population in a large census block into much smaller grid cells, using auxiliary information such as land use, road density, terrain, and others. However, in urban areas the LandScan Global^™^ data used in this study may have a lower spatial resolution than the census block. In addition, the LandScan data only give the number of people in each grid cell, without further demographic information (e.g., age and sex). Following Shi [18,19], we integrated the LandScan^™^ data and Census data to generate two raster data layers for the two age categories used in this study (15–39 and 40–49 years), respectively. We set the cell size of the resulting raster layers to be 100 m, a choice that balances spatial resolution and data volume. 

## 3. Method

### 3.1. Disaggregation

**Figure 1:**
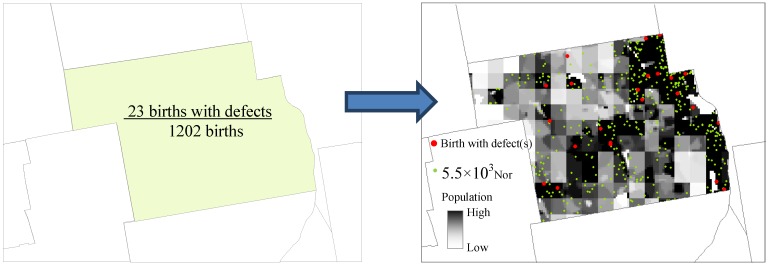
Disaggregation using the restricted and controlled Monte Carlo process.

Instead of directly using the available aggregate data to generate conventional polygon maps, we first disaggregated the data to the pixels, an approximated individual level, and then applied analytical processes designed for individual data. Our disaggregation process was a natural extension of the *Restricted and Controlled Monte Carlo* (RCMC) approach proposed in [18,19]. In our process, first, each birth from the all-birth set was assigned a random location. The randomization, however, was restricted by the town polygon that the birth fell into and controlled by the detailed population data layer described earlier. For example, for a birth with mother’s age = 35 years, we first selected the polygon of the town indicated in the data of the birth, and then referred to the raster layer of females aged 15–39 years and assigned the birth to one of the cells that are within the town polygon. The probability of a cell to receive this birth was proportional to the cell value, *i.e*., the number of women aged 15–39 years in that cell. Second, when all the births were assigned to approximated individual locations (pixels), within each town we randomly picked a number of births as the births with defects, according to the number of births with defects in that age category that occurred in the town. In this way, all the births with defects also received their approximated individual locations. Running the process again may put a birth to a different location. The difference between iterations reflects the spatial uncertainty caused by aggregation. To quantitatively assess this uncertainty and its impact on the analysis results, we ran this assigning process many times (e.g., 50 times) and generated many sets of disaggregated birth locations. Spatial analysis was then applied to each set of these locations. This RCMC process has been used in previous studies to deal with P. O. Box addresses [18,19], which only accounted for a proportion of the patient dataset. In this study, we used it to disaggregate datasets that are entirely at an aggregate level. Furthermore, we did not only disaggregate the patient (birth defect) data, but also the cohort (all-birth) data. Figure 1 illustrates the output from RCMC.

### 3.2. Intensity Estimation

**Figure 2:**
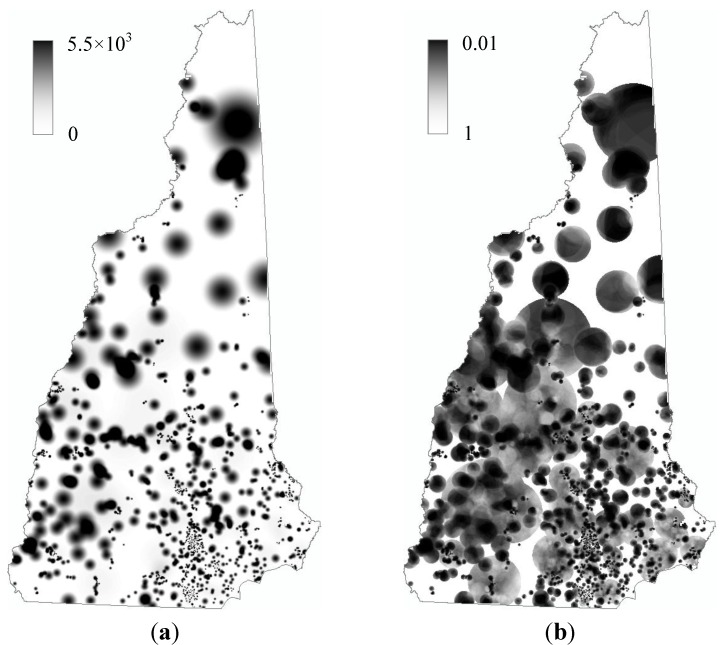
Spatial distribution of (**a**) birth defect intensity, and (**b**) its *p-value* in New Hampshire.

The disaggregated locations were then used to map the intensity of birth defects in NH. A commonly used mapping technique for individual locations is the kernel density estimation or KDE [24,25,26], which has been reviewed in [19,27]. Openshaw’s geographic analysis machine is essentially based on this method [28,29,30,31,32]. The widely used spatial filtering method is a special case of KDE [4]. Recent examples of using or testing KDE in disease mapping include [33,34,35,36,37,38]. For disease mapping, KDE must take into account the *background* of the disease, which in our specific case is all births. Essentially, what we intend to map is the local ratio between the disease and the background, and thus we call the process *Kernel Ratio Estimation* or KRE. Shi distinguishes four types of KRE, including the site-side-fixed-bandwidth, site-side-adaptive-bandwidth, case-side-fixed-bandwidth, and case-side-adaptive-bandwidth [27], and justifies the appropriateness of the case-side-adaptive-bandwidth method in disease mapping [19,27], which is what we adopted in the current study. With this method, the bandwidth of the kernel is determined by the number of births enclosed by the kernel, *i.e.*, the bandwidth adapts to the local situation of the birth distribution rather than simply use a fixed geographic distance. The kernel would be set around each birth defect case (*i.e*., case side) rather than at each grid cell for which the intensity value is to be calculated (*i.e*., site side). Over an inhomogeneous background, the case-side kernel and site-side kernel may generate rather different results. Shi [27] argues that the case-side adaptive kernel better reflects the relationship between a disease case and its *background* *support* (in our case, this *support* refers to the number of births from which a birth with defect emerges). The case-side kernel also allows a straightforward and objective way to determine a reasonable threshold for the adaptive bandwidth, which is always the greatest challenge in KDE. When the kernel is set at a disease case, it is reasonable to make the kernel to enclose just the amount of *background* that would support one case [19], which can be calculated from a standard disease/background ratio. For example, since the rate of births with defects for the age category of 15–39 years is 0.0145 (Table 1), it can be calculated that for this age category the overall prevalence of birth defects is one in 69 in NH, and thus the kernel should be set to enclose 69 births around a birth with defect(s). Each category has its own kernel threshold, and the results of the two categories were then integrated through a weighted combination following *indirect standardization* [1]. The output of this entire process is a raster layer, with the cell value representing the intensity of birth with defect(s) across NH (Figure 2(a)).

### 3.3. Statistical Significance and Spatial Uncertainty

The intensity value at a grid cell calculated by KRE needs to be evaluated for its statistical significance. This was done through a Monte Carlo process. Specifically, we let the computer randomly select births with defects from all births, not being restricted by the number of births with defects in each town. Similar processes for generating H_0_ scenarios have been widely used, and a recent example is [22]. The result of this selection was a simulated random distribution of births with defects, and was used to run the KRE analysis and generate a new raster layer of intensity. We ran this simulation 99 times to generate 99 raster layers of random distribution of intensity. Each cell value in Figure 2(a) was then compared with the 99 simulated values at the same cell. Its rank in the 100 values was considered as its *p-*value. For example, if a cell value in Figure 2(a) is the second highest among the 100 values at the same cell, it can be considered that the probability for such a high value to occur at this location is not greater than 0.02; in other words, its *p* = 0.02. The output from this analysis is a map of *p-*value (Figure 2(b)). 

**Figure 3:**
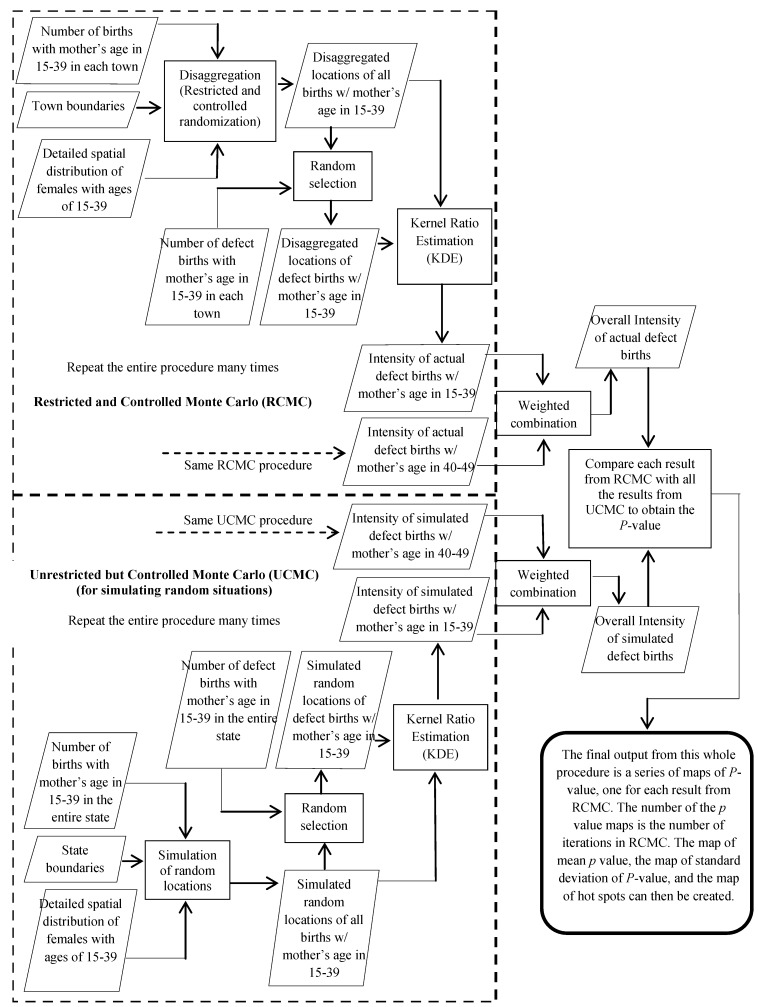
The procedure of disease mapping using the *Restricted and Controlled Monte Carlo* (RCMC): using birth defect mapping for New Hampshire as an example.

We are aware that 99 is a relatively small number of simulations to define the *p*-value(s). The estimated *p*-value will be more accurate with more simulations. The current small number of iterations is solely a result of the limited computing capacity of the desktop PC the program currently runs on. We are working on migration of the program to a high-performance computing platform.

The uncertainty sourced from aggregation can be quantified by measuring the variance in the results from different disaggregation iterations. We ran the disaggregation process 50 times and generated 50 sets of locations of births in NH. Each of the 50 sets of locations were used to run the mapping process described above, and the whole process is illustrated in Figure 3.

## 4. Results

**Figure 4:**
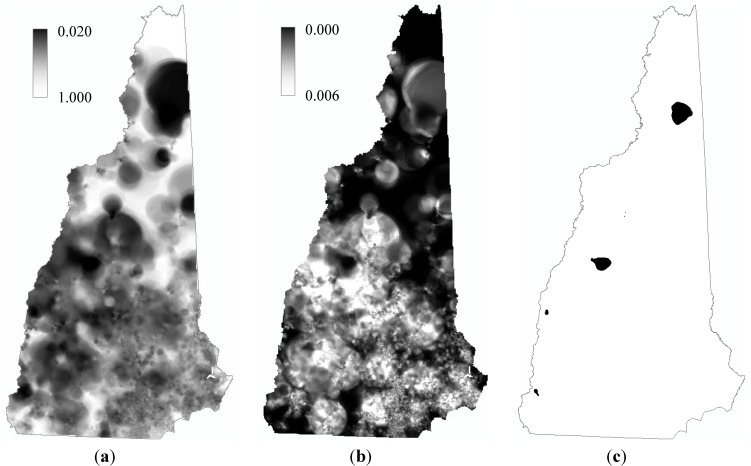
Mapping the birth defects in New Hampshire, 2003–2009. (**a**) Map of mean *p-*value; (**b**) Map of standard deviation; and (**c**) “Hot spots” identified with *α* = 0.1.

The operations with the 50 sets of disaggregated locations generated 50 *p*-value maps like that in Figure 2(b). From the 50 *p*-value maps, we generated three maps as the final output of the entire mapping process, including a map of mean *p* value, a map of standard deviation of *p-*value, and a map of *hot spots* of birth defects in NH (Figure 4). 

In the map of mean, the cell value is the simple average of the 50 *p-*values at that cell from the 50 *p*-value maps, and thus this map can be considered displaying the *representative* spatial distribution of the risk of birth defects estimated from the prevalence data; the smaller the mean *p* value, the more statistically significant the risk. In the map of standard deviation, the cell value is the standard deviation of the 50 *p-*values at that location, which can be considered as a representation of the uncertainty, caused by data aggregation, in the estimated risk value at that location, and thus this map shows the spatial distribution of the uncertainty sourced from data aggregation. The map of *hot spots* was generated by marking those grid cells whose intensity values are significantly high and the spatial uncertainties are sufficiently low. In this study, if a cell’s mean *p-*value plus two corresponding standard deviations is still smaller than 0.1 (*i.e*., α = 0.1), the cell was marked. Through this process, we identified a number of hot spots for birth defects in NH (Figure 4(c)). A more formal interpretation of a marked cell in the hot spot map is that the chance for a location to have such a high intensity of births with defects simply because of the number of births (weighted by age groups) in the location’s neighborhood is less than 10%, and the chance for a location to have such a significance of intensity simply because of the randomization in the disaggregation is beyond two standard deviations.

## 5. Discussion and Conclusions

By disaggregating the town level data through the *restricted and controlled Monte Carlo* (RCMC) process, we generated maps of birth defects for New Hampshire at the pixel level rather than the town level. RCMC is essentially a dasymetric process that allocates the total amount for an area unit to different places within the unit, by taking into ancillary information. However, conventional dasymetric mapping is deterministic, while RCMC is stochastic. Several advantages of such a mapping process can be identified:

(1) *The disaggregation allows analytical processes designed for individual data to be applied*, which avoids or mitigates the problems associated with aggregate data.
(2) *The resulting raster maps have resolutions at the pixel level (100 m in this study)*, which presents more detailed spatial distribution of disease, compared with the conventional polygon map. Those details give the raster maps advantage in detecting spatial associations between birth defects and certain environmental factors.
(3) *The RCMC process maximizes the use of available spatial information*. First of all, restricting the randomization with the smallest aggregate units maximizes the use of the spatial information represented by the polygon. Furthermore, controlling the randomization with the *background* data layer provides an open mechanism ready to take into account any available information that can help reduce spatial uncertainty and improve analysis quality. In this study, the *background* data layer of females in a certain age category eventually incorporates rich information from different sources, including the total number of people from the LandScan data and age and sex information from the Census data. The LandScan data are a product of a sophisticated model that incorporates information about population, land use, terrain, night lights, traffic, and others [39,40]. Other information, if available, can find its way into the background layer used by RCMC. For example, if a socioeconomic factor is known to be a confounding factor of a disease, and detailed information about its spatial distribution is available, it can be incorporated into the background layer.
(4) *The RCMC process explicitly quantifies the spatial uncertainty caused by data aggregation*. Little, if any, information about the spatial uncertainty in a polygon map can be conveyed to the user of the map. RCMC resolves this problem by running the randomization iteration many times. The variance in the results from these iterations represents the uncertainty caused by aggregation, which can be explicitly and easily quantified. Essentially, this is an approach based on the idea of sensitivity analysis that empirically models variance through intensive computation.

It should be particularly noted that RCMC is a stochastic process and therefore its results should not be interpreted in a deterministic way. Specifically, one should keep in mind that a value in map *a* in Figure 4 is the mean of many possible *p*-values at that location, and is not necessary the true *p*-value.

It should also be noted that the spatial uncertainty represented and presented by RCMC is only the uncertainty resulting from spatial aggregation. There are other spatial uncertainties in the result, such as that from KRE. The bandwidth of kernel is eventually an instrument and representation of spatial uncertainty: the larger the bandwidth, the higher the spatial uncertainty. Specifically, if we consider a disease case to be a realization of a random variable in its *support* (*i.e*., the population at risk around the disease case), then the more extensively the support is geographically distributed, the more uncertain where that realization will occur. A background-adaptive bandwidth (the one used in this study) may become fairly large in a less populous area to enclose enough support, in order to ensure statistical stability of the estimated ratio value. In other words, in a less populous area, the means for maintaining statistical stability is to increase the spatial uncertainty. More generally, usually the spatial uncertainty and statistical stability form a tradeoff [19]. Therefore, a large dark patch in the hot spot map, like the one in north NH in Figure 4(c), does not necessarily mean that the entire area is a high risk region. The proper interpretation of such large patches is that there are high-risk locations within these areas.

Like many previous methods of its kind, this process has an inherent problem of multiple testing. If the test at each cell is independent from one another, under α = 0.1 it is expected to see 10% of the cells standing out as significant, even though they may not bear epidemiological meaning. In fact, the marked cells in our hot spot map only account for 1% of all the cells in NH, which makes it possible that they are simply an outcome of multiple testing. However, it should be considered that: (1) the test at each cell is not independent, as the kernel at a cell has substantial overlap with its nearby kernels [31,32]; and (2) the constraint applied to the RCMC output (*i.e*., the two-standard deviation cut) sets a high bar for a cell to be marked as a hot-spot cell. Both should have considerably mitigated the problem of multiple testing, although to quantitatively evaluate their effects is complicated and yet to be explored. After all, the primary goal of disease mapping is to help form hypotheses and inform research design for further investigations, rather than draw determinant conclusions. Even it does not eliminate the problem of multiple testing, the proposed method is advantageous over the conventional mapping methods based on aggregate data in serving this exploratory purpose. 
